# Supplementary material for: Geographic distribution of hospice, homecare, and nursing home facilities and access to end-of-life care among persons living with HIV/AIDS in Appalachia
Source: PLoS One. 2020 Dec 14;15(12):e0243814. doi: 10.1371/journal.pone.0243814 (PMC7735579; doi:10.1371/journal.pone.0243814)
Supplement: Attachment — Submitted filename: Response to Reviewer Comments_11_27_20.docx [file pone.0243814.s002.docx]

**Response to Review Comments**

**Editor’s Comment**

1. Please remove 'd' from the end of 'advance' in 'advance care planning' throughout. This is a common mistake, but one that PLOS ONE should not be perpetuating.

**Response**

The manuscript has been revised to remove the ‘d’ in advanced care planning to now read, “advance care planning.” I have included a clean copy and tracked changes copy.
