# Supplementary material for: Geographic distribution of hospice, homecare, and nursing home facilities and access to end-of-life care among persons living with HIV/AIDS in Appalachia
Source: PLoS One. 2020 Dec 14;15(12):e0243814. doi: 10.1371/journal.pone.0243814 (PMC7735579; doi:10.1371/journal.pone.0243814)
Supplement: Attachment — Submitted filename: Response to Reviewer Comments.docx [file pone.0243814.s001.docx]

**Response to Review Comments**

**Editor’s Comments**

**Editor’s Comment**

1. Please ensure that your manuscript meets PLOS ONE's style requirements, including those for file naming. The PLOS ONE style templates can be found at

https://journals.plos.org/plosone/s/file?id=wjVg/PLOSOne_formatting_sample_main_body.pdf and

https://journals.plos.org/plosone/s/file?id=ba62/PLOSOne_formatting_sample_title_authors_affiliations.pdf

**Response**

The manuscript has been revised to meet all of PLOS ONE’s style requirements.

**Editor’s Comment**

2. Please include in your Methods section the date ranges over which you recruited participants to this study.

**Response**

The date ranges for the study have been added to the “Data Collection” sub-section of the “Methods” section of the manuscript.

**Editor’s Comment**

3. Please include additional information regarding the survey or questionnaire used in the study and ensure that you have provided sufficient details that others could replicate the analyses. If you developed and/or translated a questionnaire as part of this study and it is not under a copyright license more restrictive than Creative Commons Attribution (CC-BY), please include a copy, in both the original language and English, as Supporting Information.

**Response**

- We have added additional information about the survey questionnaire including when it was done as well as information on pre-testing of the questionnaire. This information was added to the “data collection” sub-section of the “methods” section.
- We have included the questionnaire in the revised submission.

**Editor’s Comment**

4.We note that you have indicated that data from this study are available upon request. PLOS only allows data to be available upon request if there are legal or ethical restrictions on sharing data publicly. For more information on unacceptable data access restrictions, please see http://journals.plos.org/plosone/s/data-availability#loc-unacceptable-data-access-restrictions.

In your revised cover letter, please address the following prompts:

a) If there are ethical or legal restrictions on sharing a de-identified data set, please explain them in detail (e.g., data contain potentially sensitive information, data are owned by a third-party organization, etc.) and who has imposed them (e.g., an ethics committee). Please also provide contact information for a data access committee, ethics committee, or other institutional body to which data requests may be sent.

b) If there are no restrictions, please upload the minimal anonymized data set necessary to replicate your study findings as either Supporting Information files or to a stable, public repository and provide us with the relevant URLs, DOIs, or accession numbers. For a list of acceptable repositories, please see http://journals.plos.org/plosone/s/data-availability#loc-recommended-repositories.

We will update your Data Availability statement on your behalf to reflect the information you provide.

**Response** De-identified study data was uploaded to Dryad as part of this resubmission and will be released upon publication of the manuscript.

The DOI for the dataset is as follows:

Hutson, Sadie (2020), End of Life Care Among Persons with HIV, Dryad, Dataset, <https://doi.org/10.5061/dryad.t1g1jwt13>

**Comment**

5. Please include a separate caption for each figure in your manuscript.

**Response**

Figure captions of all the manuscript figures have been added to the end of the manuscript text.

**Comment**

6. Please include your tables as part of your main manuscript and remove the individual files. Please note that supplementary tables (should remain/ be uploaded) as separate "supporting information" files.

**Response**

The table (Table 1) has been included as part of the manuscript. The original individual files has been removed from the submission.

**Comment**

7.We note that [Figure(s) 1, 2, 3 and 4] in your submission contain [map/satellite] images which may be copyrighted. All PLOS content is published under the Creative Commons Attribution License (CC BY 4.0), which means that the manuscript, images, and Supporting Information files will be freely available online, and any third party is permitted to access, download, copy, distribute, and use these materials in any way, even commercially, with proper attribution. For these reasons, we cannot publish previously copyrighted maps or satellite images created using proprietary data, such as Google software (Google Maps, Street View, and Earth). For more information, see our copyright guidelines: http://journals.plos.org/plosone/s/licenses-and-copyright.

We require you to either (1) present written permission from the copyright holder to publish these figures specifically under the CC BY 4.0 license, or (2) remove the figures from your submission:

1. You may seek permission from the original copyright holder of Figure(s) [1, 2, 3 and 4] to publish the content specifically under the CC BY 4.0 license.

We recommend that you contact the original copyright holder with the Content Permission Form (http://journals.plos.org/plosone/s/file?id=7c09/content-permission-form.pdf) and the following text:

“I request permission for the open-access journal PLOS ONE to publish XXX under the Creative Commons Attribution License (CCAL) CC BY 4.0 (http://creativecommons.org/licenses/by/4.0/). Please be aware that this license allows unrestricted use and distribution, even commercially, by third parties. Please reply and provide explicit written permission to publish XXX under a CC BY license and complete the attached form.”

Please upload the completed Content Permission Form or other proof of granted permissions as an "Other" file with your submission.

In the figure caption of the copyrighted figure, please include the following text: “Reprinted from [ref] under a CC BY license, with permission from [name of publisher], original copyright [original copyright year].”

2. If you are unable to obtain permission from the original copyright holder to publish these figures under the CC BY 4.0 license or if the copyright holder’s requirements are incompatible with the CC BY 4.0 license, please either i) remove the figure or ii) supply a replacement figure that complies with the CC BY 4.0 license. Please check copyright information on all replacement figures and update the figure caption with source information. If applicable, please specify in the figure caption text when a figure is similar but not identical to the original image and is therefore for illustrative purposes only.

The following resources for replacing copyrighted map figures may be helpful:

USGS National Map Viewer (public domain): http://viewer.nationalmap.gov/viewer/

The Gateway to Astronaut Photography of Earth (public domain): http://eol.jsc.nasa.gov/sseop/clickmap/

Maps at the CIA (public domain): https://www.cia.gov/library/publications/the-world-factbook/index.html and https://www.cia.gov/library/publications/cia-maps-publications/index.html

NASA Earth Observatory (public domain): http://earthobservatory.nasa.gov/

Landsat: http://landsat.visibleearth.nasa.gov/

USGS EROS (Earth Resources Observatory and Science (EROS) Center) (public domain): http://eros.usgs.gov/#

Natural Earth (public domain): http://www.naturalearthdata.com/

**Response**

The required copyright license has been attached as “Other” file together with the revised manuscript

**Reviewers' comments:**

**Reviewer #1:**

**Comment**

This is an interesting paper and highlights some startling facts about the prevalence of HIV/Aids in this geographical region and the provision available for EOL care.

The data presented are detailed, but difficult for an international audience to easily grasp. The problems addressed are very real. But I think a more detailed theoretical and literature based treatment of the data would be helpful.

I think this is an important public health issue, but wonder of PLOS One is the right place for this to appear. I would hope that you might be able to draw the attention of local public and private service planners in your area. I realise that the group you are looking at is unlikely to be able to access health insurance and so their situation is of even more importance for health providers in USA to consider.

It would be good to know how these matters are addressed in other parts of the USA where there must be a number of isolated communities and people facing similar social and demographic difficulties in managing their care.

**Response**

The discussion and background sections of the manuscript have been revised to address the reviewers’ comments. In addition, we thoroughly reviewed the revised document and made several revisions (in addition to those requested by the editor and reviewers) to improve clarity and flow.

**Reviewer #2:**

**Comment**

Overall this paper does a good job of illustrating unequal access to hospice and palliative care in rural areas of the SE United States.

I do have a couple of comments and observations that will make this paper stronger especially from a geographic perspective.

First the literature review section should include a review of other methods to calculate travel time - given that travel time is the deciding variable for assessing accessibility.

They could start with these papers:

1. Tansley G, Schuurman N, Bowes M, Erdogan M, Asbridge M, Yanhar N. Effect of predicted travel time to trauma care on mortality in major trauma patients in Nova Scotia. Canadian Journal of Surgery. 2018;Accepted August 28, 2018.

2. Schuurman N, Fiedler R, Grzybowski S, Grund D. Defining rational hospital catchments for non-urban areas based on travel-time. International Journal of Health Geographics. 2006;5(43).

3. Walker BB, Schuurman N, Auluck A, Lear AS, Rosin M. Socioeconomic Disparities in Head and Neck Cancer Patients’ Geographical Access to Comprehensive Cancer Treatment Centres in British Columbia, Canada. Rural and Remote Health

. 2017.

4. Tansley G, Schuurman N, Amram O, Yanchar N. Spatial access to emergency services in low- and middle-income countries: a GIS-based analysis. PLOS One. 2015.

Basically to calculate travel time, you can use origin destination matrices. Other methods such as the network analysis described here are totally acceptable but there should be some discussion of relative efficacy.

Finally the figures are (in my file anyway) totally pixelated and unreadable.

**Response**

We thank the reviewer for raising this issue that would help to improve the strength of the paper from geographic perspective. As per the reviewer’s recommendation, we have added (to the background section) review of methods for calculation of travel time. We have used and cited all the articles recommended by the reviewer as well as other relevant bodies of work. We greatly appreciate the reviewer’s thoughtful review and recommendation.

Regarding the digital quality (resolution) of the figures, we suspect the reviewer must have used the low-resolution versions of figures during the review. High resolution figures have been uploaded and they look fine on the copies submitted.
